# Increasing temperature-driven changes in life history traits and gene expression of an Antarctic tardigrade species

**DOI:** 10.3389/fphys.2023.1258932

**Published:** 2023-09-12

**Authors:** Ilaria Giovannini, Chiara Manfrin, Samuele Greco, Joel Vincenzi, Tiziana Altiero, Roberto Guidetti, Piero Giulianini, Lorena Rebecchi

**Affiliations:** ^1^ Department of Life Sciences, University of Modena and Reggio Emilia, Modena, Italy; ^2^ NBFC, National Biodiversity Future Center, Palermo, Italy; ^3^ Department of Life Sciences, University of Trieste, Trieste, Italy; ^4^ Department of Education and Humanities, University of Modena and Reggio Emilia, Reggio Emilia, Italy

**Keywords:** global warming, thermal stress, life cycle, fitness, transcriptome, DEGs (differentially expressed genes), TDPs (tardigrade disordered proteins), Antarctica

## Abstract

The Antarctic region has been experiencing some of the planet’s strongest climatic changes, including an expected increase of the land temperature. The potential effects of this warming trend will lead ecosystems to a risk of losing biodiversity. Antarctic mosses and lichens host different microbial groups, micro-arthropods and meiofaunal organisms (e.g., tardigrades, rotifers). The eutardigrade *Acutuncus antarcticus* is considered a model animal to study the effect of increasing temperature due to global warming on Antarctic terrestrial communities. In this study, life history traits and fitness of this species are analyzed by rearing specimens at two different and increasing temperatures (5°C vs. 15°C). Moreover, the first transcriptome analysis on *A. antarcticus* is performed, exposing adult animals to a gradual increase of temperature (5°C, 10°C, 15°C, and 20°C) to find differentially expressed genes under short- (1 day) and long-term (15 days) heat stress. *Acutuncus antarcticus* specimens reared at 5°C live longer (maximum life span: 686 days), reach sexual maturity later, lay more eggs (which hatch in longer time and in lower percentage) compared with animals reared at 15°C. The fitness decreases in animals belonging to the second generation at both rearing temperatures. The short-term heat exposure leads to significant changes at transcriptomic level, with 67 differentially expressed genes. Of these, 23 upregulated genes suggest alterations of mitochondrial activity and oxido-reductive processes, and two intrinsically disordered protein genes confirm their role to cope with heat stress. The long-term exposure induces alterations limited to 14 genes, and only one annotated gene is upregulated in response to both heat stresses. The decline in transcriptomic response after a long-term exposure indicates that the changes observed in the short-term are likely due to an acclimation response. Therefore, *A. antarcticus* could be able to cope with increasing temperature over time, including the future conditions imposed by global climate change.

## Introduction

Industrial, agricultural and livestock activities have been identified as drivers of the changes in atmospheric composition, providing evidence supporting the influence of human activities on global warming (IPCC, 2018). The global mean temperature of Earth surface has increased by 1.09°C over the last 150 years, and it is projected to warm by 1.0°C–5.7°C by 2100, in relation to the emissions of greenhouse gases (Chown et al., 2022). Especially, the Antarctic region has been experiencing some of the planet’s strongest climatic changes, including extreme climate and weather events, droughts, floods, accelerated ice loss, increased glacier and ice sheet meltwater, ocean acidification and changes in mean sea level (Golledge et al., 2019; Edwards et al., 2021; Chown et al., 2022; Constable et al., 2022). The land temperature increased to 0.61°C ± 0.34°C per decade between 1989 and 2018 (IPCC, 2018; Robinson, 2022), while the ocean is experiencing the greatest absolute oxygen loss (Bronselaer et al., 2020). Moreover, significant stratospheric ozone depletion persists over Antarctica during the austral spring season, even though evidence suggests that the hole in the ozone layer over Antarctica is showing signs of recovery since the year 2000 (IPCC, 2021). The increased frequency, severity, and duration of extreme events in the short term, will lead many terrestrial, freshwater, coastal, and marine ecosystems to a very high or high risk of losing biodiversity (IPCC, 2022). Antarctic ecosystems have significantly lower biodiversity than the rest of the planet (Convey and Stevens, 2007). Life on the land is mostly restricted to the small ice-free areas of the continent (<0.5% by total area; Chown et al., 2022); mosses and lichens are common, and include different microbial groups (prokaryotes, algae, fungi, and protists), micro-arthropods (mites, springtails, and midges) and micro-invertebrates of the meiofauna (i.e., nematodes, rotifers, and tardigrades; Câmara et al., 2021; Phillips et al., 2022). Nevertheless, switches between dominant and least represented communities for both moss communities and invertebrate species have been recently evidenced (Chown et al., 2022). The primary factors influencing the abundance, composition, and distribution of Antarctic terrestrial biodiversity are temperature, liquid water and nutrient availability (Kennedy, 1993; Convey, 1996; Hogg et al., 2006; Sinclair et al., 2006; Bissett et al., 2014; Chown and Convey, 2016). However, recent evidence indicates an important role of biotic interactions within and between species in shaping Antarctic communities (Potts et al., 2020).

Due to the ongoing trend of increasingly warmer summers and more frequent warming events, the effects of climate change on Antarctic biodiversity are becoming progressively clear in the transformation, damage, and degradation of ecosystems (Andriuzzi et al., 2018; IPCC, 2022). To expand the knowledge about the effects of global warming on Antarctic meiofauna, we investigated the adaptive responses of the Antarctic tardigrade *Acutuncus antarcticus* (Richters, 1904) to increasing temperature. We analyzed and compared its life history traits at different temperatures (5°C vs. 15°C), and we performed a transcriptome analysis to investigate the molecular mechanisms involved in heat response and tolerance of this species. *A. antarcticus* is the most abundant and common eutardigrade in Antarctica (Cesari et al., 2016) inhabiting freshwater ecosystems and terrestrial soils, mosses, and lichens (Murray, 1910; Dastych, 1991; Cesari et al., 2016). It is considered to be a parthenogenetic pan-Antarctic species and it is herbivorous and bacteriophagous (Altiero et al., 2015; Cesari et al., 2016). Previous studies demonstrated that it is able to tolerate dehydration, freezing, short exposure (1 h) to high temperatures (up to 37°C), and exposure to UV radiation (Giovannini et al., 2018). Since it colonizes different habitats, it could be considered as a representative animal model for Antarctic terrestrial communities.

## Materials and methods

### Sampling and rearing

Specimens of *A. antarcticus* were extracted from bottom sediments of a temporary freshwater pond close to the Italian Antarctic base “Mario Zucchelli” in Victoria Land (125 m a.s.l., 74°42.5800 S, 164°06.0860 E, Terranova Bay, Antarctica) and used to create rearing microcosms at two different temperatures (5°C and 15°C), as described in Altiero et al. (2015). The microcosms were kept at photoperiod 12 h/12 h (L/D) and maintained in several flasks with algal culture medium in spring mineral water (volume ratio 1:3) and unicellular alga *Chlorococcum* sp. as a food source at the laboratory of Evolutionary Zoology of University of Modena and Reggio Emilia (Italy).

### Life history traits

To collect data on life history traits of *A. antarcticus*, 10 adult females with oocytes in the gonad were collected from the microcosms and defined as the parental generation (P). These animals were individually cultured for 141 days at 5°C with a photoperiod 12 h/12 h (L/D) as indicated in Altiero et al. (2015). Laid eggs were isolated until hatching and the newborns were individually reared from birth to death and kept at the same laboratory conditions. The offspring of the parental females represented the successive filial (F_1_ and F_2_) generations.

For animals belonging to F_1_ and F_2_ generations, data on active life span, number of molts, age at first oviposition, number of ovipositions per life span, number of laid eggs per life span (fecundity), number of eggs per clutch (fertility), interval of time between ovipositions, egg hatching time and egg hatching percentage were collected (Supplementary files S1–S3). The reproductive features analyzed for the P generation were: interval of time between ovipositions, egg hatching time and hatching percentage (Supplementary files S1–S3).

### Statistical analysis

A statistical comparison of each life history trait among generations was carried out. The comparisons of each life history trait between the rearing temperature of 5°C (obtained in present study) and the rearing temperature of 15°C [data obtained in a previous study by Altiero et al. (2015)] were also performed to evaluate the effect of increasing temperature on each life history trait. Statistical analyses were performed using R (R Core Team, 2020; Supplementary file S4). Generalized linear models (GLM) were carried out to test the effects of the generation, the increasing temperature, and their interaction on each life history trait. In addition, the effect of the order of oviposition on fertility was evaluated, as well as the effect of egg number per clutch on egg hatching time and on egg hatching percentage. Reports of these GLM models were generated with the R package “report” (Makowski et al., 2020; Supplementary Table S1). Standardized parameters were obtained by fitting the GLM models on a standardized version of the dataset. The 95% Confidence Intervals (CIs) and *p*-values were computed using a Wald z-distribution approximation.

### Fitness formula

A fitness score was calculated for each female belonging to both F_1_ and F_2_ generations and reared at 5°C or 15°C. Parameters considered to calculate fitness score were those relevant to contribute to future generations: early age at first oviposition, short hatching time, high number of laid eggs (fecundity) and high percentage of hatched eggs. Fecundity and total hatched eggs were normalized in range 0–1 using the min-max normalization formula:

$$=\frac{x-\min\;\left(\underline{x}\right)}{\max\left(\underline{x}\right)-\min\;\left(\underline{x}\right)}$$

The number of hatched eggs was considered the fundamental requirement to contribute to successive generations and a fitness score of zero was attributed to females that never laid eggs or whose laid eggs did not hatch.

The hatching time and the age at first oviposition were normalized in range 0–1 using the formula:

$$=1-\frac{x-\min\;\left(\underline{x}\right)}{\max\left(\underline{x}\right)-\min\;\left(\underline{x}\right)}$$

The fitness scores are obtained by calculating a mean of the four values previously normalized. The data were analyzed using the program Google Colab (in Python) and the libraries Pandas, Seaborn and Matplotub. The fitness scores were statistically compared among generations and rearing temperatures with the non-parametric Kruskal-Wallis test using the SPSS 28 program.

### Expression library preparation

**FIGURE 1:**
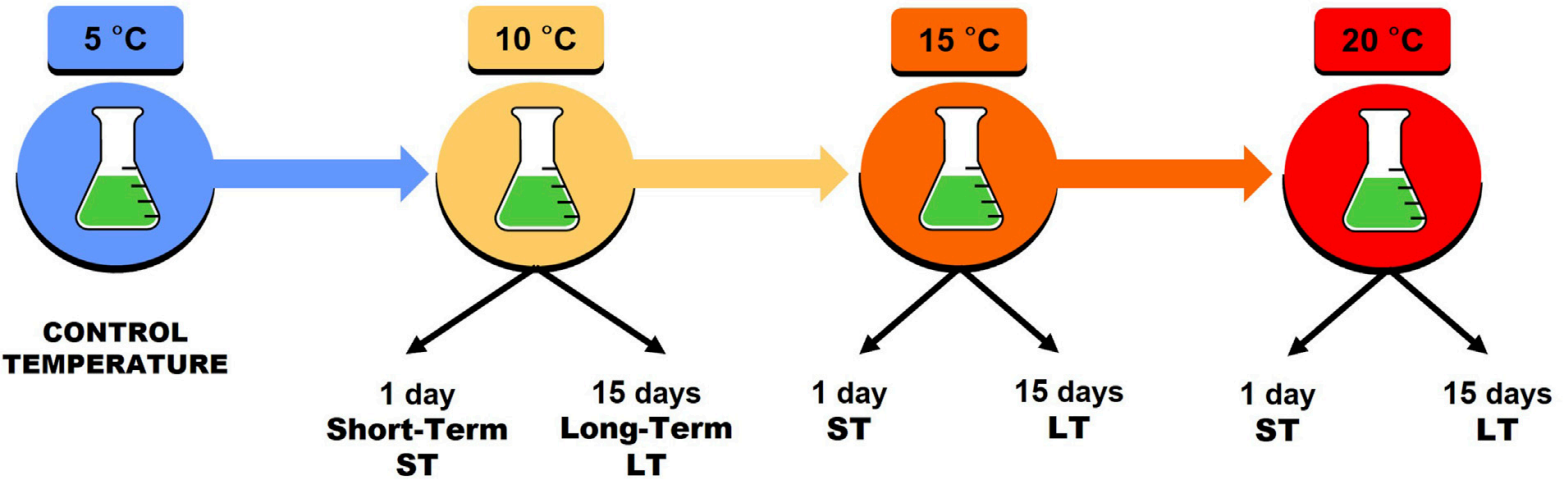
Experimental protocol. Temperatures tested: 5°C (control temperature), 10°C, 15°C, and 20°C. Thermal stress tested: ST short-term (1 day of exposure) and LT long-term (15 days of exposure). ST and LT were planned for 10°C, 15°C, and 20°C. Non-step shifts were performed from one temperature to the next every 15 days.

The experimental protocol used for the setting-up of transcriptomic libraries involved sequential increments of 5°C of the incubation temperature. The rearing microcosms, initially maintained at 5°C (control temperature), were then raised to 10°C, then to 15°C, and finally to the highest tested temperature of 20°C. The shift from one temperature to the next one was performed every 15 days (Figure 1). Transcriptomic analysis was conducted at two different time-point stresses for each incubation temperature: 1 day and 15 days after the start of exposure to the stress-inducing incubation temperature. Exposure after 1 day simulates short-term heat stress (ST), while exposure after 15 days simulates long-term heat stress (LT). For each thermal increase step (10°C, 15°C, and 20°C), as well as for both ST and LT stress conditions, 3 pools of 100 active animals were utilized for RNA extraction. As an experimental control, RNA was isolated from three replicates of 100 active animals each that were collected from the microcosms at 5°C.

Before being subjected to RNA extraction, tardigrades of each pool were kept starved in rearing water for 24 h at the assayed experimental temperature. At the end of this period, inactive tardigrades were replaced with live specimens from a reserve pool kept in the same conditions. Any laid eggs and exuviae were also removed from each microtube.

For the RNA extractions, tardigrades were placed in microtubes from which as much water as possible was removed. RNA extractions were performed using the Tissue-ruptor, a tissue homogenizer, and the Trizol (TRI) reagent. To reduce DNA contaminations, an enzymatic digestion step was also performed, using DNAase I. RNA was retro-transcribed into cDNA using the kit SuperScript^®^ III First-Strand Synthesis System for RT-PCR (ThermoFisher Scientific). Each qPCR was performed placing in the multi-well plate the cDNA from extracted RNA, a positive control (genomic DNA of *A. antarcticus*), a negative control (no template) and the extracted RNA in order to evaluate the amplification of the 18S gene. The amplification of cDNA and no amplification of RNA indicates the absence of DNA contamination in the RNA sample. To assess the RNA integrity, a step of qualitative validation was carried out using the Agilent 2100 Bioanalyzer system.

### RNAseq data preparation

Sequencing of the RNA libraries was performed by the Genomics and Epigenomics Platform of Area Science Park (Trieste, Italy) on an Illumina^®^ NovaSeq 6000 platform with a 2 × 150 bp strategy. Raw sequencing reads were uploaded to SRA under the BioProject id PRJNA851942 for public availability (https://dataview.ncbi.nlm.nih.gov/object/PRJNA851942?reviewer=3eipk7fcuuqbppjdr0en90cq8e).

The quality of the raw reads was assessed with fastqc (Andrews, 2020) plus multiqc (Ewels et al., 2016), and trimmed according to the results with fastp v0.20 (Chen et al., 2018). Trimmed reads were merged in order to assemble a reference transcriptome using the multi-assembler strategy provided by the Oyster River Protocol (ORP) pipeline (MacManes, 2018), with the TPM_FILT parameter set to 1 and all other parameters left as default. The assembled transcriptome was quality checked with the trinityStats.pl script provided within the Trinity software package (Grabherr et al., 2011), while its completeness was assessed with BUSCO v.5 (Manni et al., 2021) against the Metazoa database of OrthoDB v.10 (Kriventseva et al., 2019). Since an exploratory analysis on the expression data showed high intra-replica variability, suggesting the presence of some level of contamination, we used published tardigrade genomes to refine it. This was achieved using blastn (Camacho et al., 2009) to query it to the reference genomes of *Hypsibius dujardini* [Delmont and Eren, 2016; Yoshida et al., 2017; redescribed as *Hypsibius exemplaris* by Gąsiorek et al. (2018)], and *Ramazzottius varieornatus* (Hashimoto et al., 2016) with a word size of 9 and e-value threshold of 1 × 10^−5^. The completeness of the refined assembly was re-evaluated with BUSCO and the contigs were functionally annotated with annot.aM available at https://gitlab.com/54mu/annotaM. To identify transcriptional isoforms within the transcriptome, the proteomes derived from the aforementioned tardigrade genomes were clustered with cd-hit v.4.8.1 (Fu et al., 2012) (-c parameter set to 0.99) and merged with the UniProt-SwissProt database. This sequence collection was then used as a database for a run of diamond (Buchfink, 2021) in blastx mode, using the transcriptome as a query. Contigs sharing the same best hit (by bitscore) were assigned to the same gene and deemed transcriptional isoforms. Although this approach does not discriminate between proper splicing isoforms and multi-copy genes, it is useful to remove noise during the Differential Gene Expression analysis. Gene Expression quantification was performed with salmon v.1.8 (Patro et al., 2017) on the individual samples, quantifying at both transcript and gene level using the count metric. Expression data was finally loaded in a R environment for Differential Gene Expression (DGE) analysis.

### Gene expression analysis

Batch effect was evaluated and removed with RUVSeq (Risso et al., 2014) with an empirical control gene list, created from the 1000 genes showing the lowest variation in gene expression. This list was obtained from a first pass of Differential Gene Expression (DGE) analysis, with an all vs. all design with edgeR (McCarthy et al., 2012). Identification (and subsequent removal) of potential outlier samples was performed by visualization of MDS and PCA plots. The normalized data was then processed in a second pass of DGE with edgeR, analyzing the two exposure times together and independently. The two exposure times were analyzed separately and jointly, and each temperature group was compared with the control group in an ANOVA-like fashion. DEGs were identified with the Generalized Linear Model built into edgeR, with a FDR corrected *p*-value significance threshold of 0.01. Gene Ontology (GO) and PFAM domain enrichment analysis was performed by hypergeometric test (Falcon and Gentleman, 2008), comparing the DEG sets with the set of all expressed genes (obtained by the filterByExpr function of edgeR). A term was deemed significantly enriched with a FDR <0.05 and an observed-expected value >3.

## Results

**FIGURE 2:**
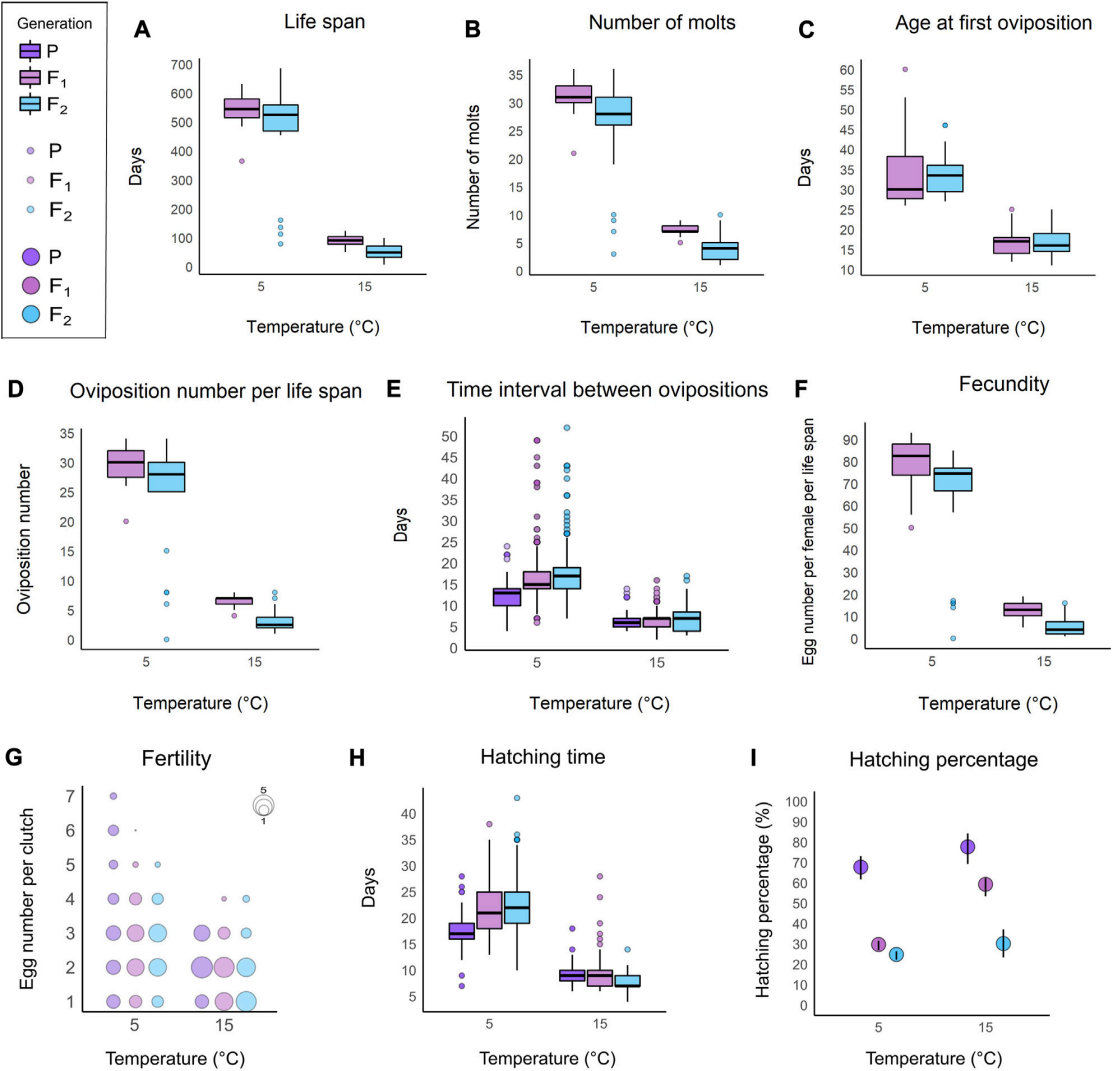
Plots comparing the life history traits of *A. antarcticus* among the three generations (P: parental; F_1_: first filial generation; F_2_: second filial generation) under different rearing temperatures (5°C and 15°C). **(A)** Active life span. **(B)** Number of molts. **(C)** Age at first oviposition. **(D)** Number of ovipositions per life span. **(E)** Interval of time between ovipositions. **(F)** Number of laid eggs per life span (fecundity). **(G)** Number of eggs per clutch (fertility). **(H)** Egg hatching time. **(I)** Hatching percentage. **A–F**; **(H)** within each box, horizontal black lines denote median values; boxes extend from the 25th to the 75th percentile of each group’s distribution of values; vertical extending lines denote adjacent values (i.e., the most extreme values within 1.5 interquartile range of the 25th and 75th percentile of each group); dots denote observations outside the range of adjacent values. **(I)** vertical black lines denote the 95% confidence interval.

The active life span of *A. antarcticus* reared at 5°C reached a maximum value of 686 days in a specimen of F_2_ generation. Adult females laid eggs freely once a fortnight, and they molted before every egg oviposition. Throughout her life span, each female laid up to 93 eggs, with a maximum of 34 ovipositions. Newborns molted once before their first oviposition at the age of about 34 days. The eggs hatched in about 22 days. Detailed collected data and performed analyses of life history traits of P, F_1_, and F_2_ generations reared at 5°C are presented in Figure 2 (Supplementary Table S2, Supplementary files S1–S3).

### GLM results

Life span, number of molts, the number of ovipositions per life span, and the number of laid eggs per life span are reduced by the effect of F_2_ generation, by the effect of increasing temperature (from 5°C to 15°C) and by the effect of the high temperature (15°C) on the F_2_ generation (Figures 2A, B, D, F; Supplementary file S5).

The age at first oviposition is decreased by the effect of increasing temperature (from 5°C to 15°C; Figure 2C; Supplementary file S5).

The time interval between ovipositions is reduced by the effect of parental generation and by the effect of increasing temperature (from 5°C to 15°C; Figure 2E; Supplementary file S5). On the other hand, this life history trait is increased by the effect of F_2_ generation and by the effect of the high temperature (15°C) on the parental generation (Figure 2E; Supplementary file S5).

The number of eggs per clutch significantly increased in relation to the order of oviposition, therefore the number of eggs per clutch increased during the life span for all animals. No effects on the number of eggs per clutch were evidenced in relation to the temperature increase (from 5°C to 15°C) and to the belonging to different generations (Figure 2G; Supplementary file S5).

The egg hatching time is reduced by the effect of parental generation, by the effect of increasing temperature (from 5°C to 15°C) and by the effect of the high temperature (15°C) on the F_2_ generation (Figure 2H; Supplementary file S5). Nevertheless, the hatching time is increased by the effect of the number of eggs per clutch and by the effect of the high temperature (15°C) on the parental generation (Figure 2H; Supplementary file S5).

The egg hatching percentage is increased by the effect of parental generation, by the effect of increasing temperature (from 5°C to 15°C) and by the effect of the number of eggs per clutch (Figure 2I; Supplementary file S5). Otherwise, the hatching percentage is reduced by the effect of F_2_ generation, by the effect of the high temperature (15°C) on the parental generation and by the effect of the high temperature (15°C) on the F_2_ generation (Figure 2I; Supplementary file S5).

### Fitness score

**FIGURE 3:**
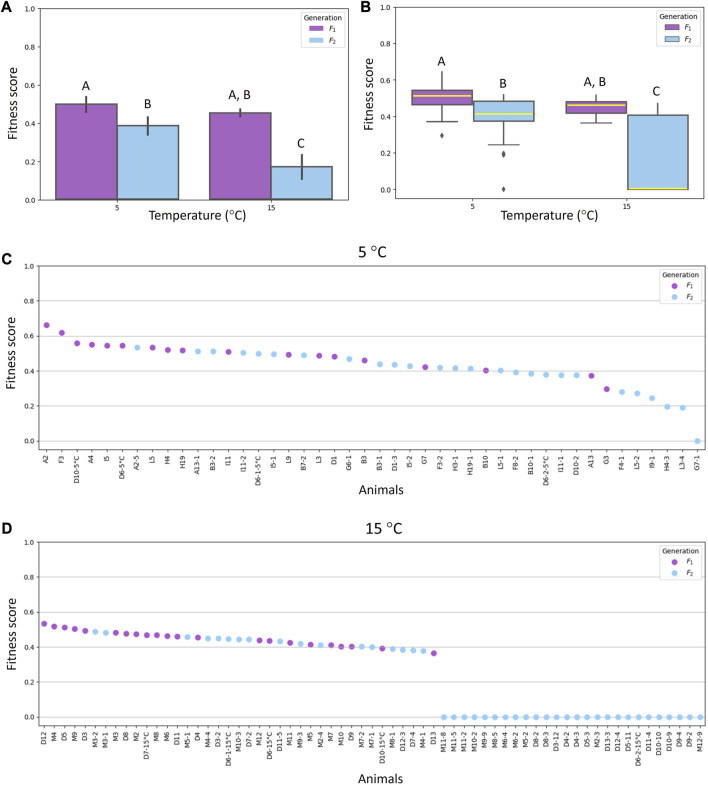
Fitness scores of *Acutuncus antarcticus* specimens belonging to F_1_ (first filial generation) and F_2_ (second filial generation) reared at different temperatures (5°C and 15°C). **(A)** Means ± standard deviation of fitness scores of specimens of both F_1_ and F_2_ generations reared at 5°C or 15°C. **(B)** Plots comparing the fitness scores of specimens of both F_1_ and F_2_ generations reared at 5°C or 15°C. Within each box, horizontal yellow lines denote median values; boxes extend from the 25th to the 75th percentile of each group’s distribution of values; vertical extending lines denote adjacent values (i.e., the most extreme values within the 1.5 interquartile range of the 25th and 75th percentile of each group); dots denote observations outside the range of adjacent values. **(C)** Individual fitness scores of specimens reared at 5°C. **(D)** Individual fitness scores of specimens reared at 15°C. **(A,B)** Different letters above columns and plots indicate significant differences between groups, whereas shared letters indicate no significant differences. B differs from A (*p* < 0.05); C differs from A and B (*p* ≤ 0.001).

Among parameters relevant to calculate fitness score, the high number of laid eggs and the high number of hatched eggs contributed positively to future generations of animals reared at 5°C, while the early age at first oviposition and the short hatching time contributed positively to future generations of animals reared at 15°C. The data obtained on fitness scores are shown in Figure 3 (Supplementary file S6). The maximum value was 0.66 and was reached by a female of F_1_ generation reared at 5°C (Figure 3C).

Fitness scores of animals belonging to F_2_ generation reared at 5°C showed statistically significant decrease (*p* < 0.05) compared with the fitness scores of animals belonging to F_1_ generation and reared at the same temperature. Moreover, a significant reduction (*p* ≤ 0.001) was recorded in the fitness scores of animals belonging to the F_2_ generation and reared at 15°C compared with other animal fitness scores (Figures 3A, B). Among animals reared at 15°C and belonging to the second generation, there were 26 females with a fitness score of zero, since 13 did not lay eggs and 13 laid eggs that never hatched (Figure 3D).

### Transcriptomic results

The overall high quality of sequencing reads (Supplementary file S7) allowed a high quality first assembly, with 79% complete, 7.7% fragmented and 13.3% missing BUSCOs compared with the Metazoa OrthoDB. The complete BUSCOs showed a high degree of redundancy, 362 out of 754 complete BUSCOs were marked as duplicated. After the removal of contaminant sequences, the BUSCO scores were unchanged.

The first part of the expression analysis evidenced a high degree of variability between samples, independent of the presence of sequences from putative contaminants. As a consequence, many samples were considered outliers and had to be removed from the analysis. As a matter of fact, the most diverse control sample was discarded.

**FIGURE 4:**
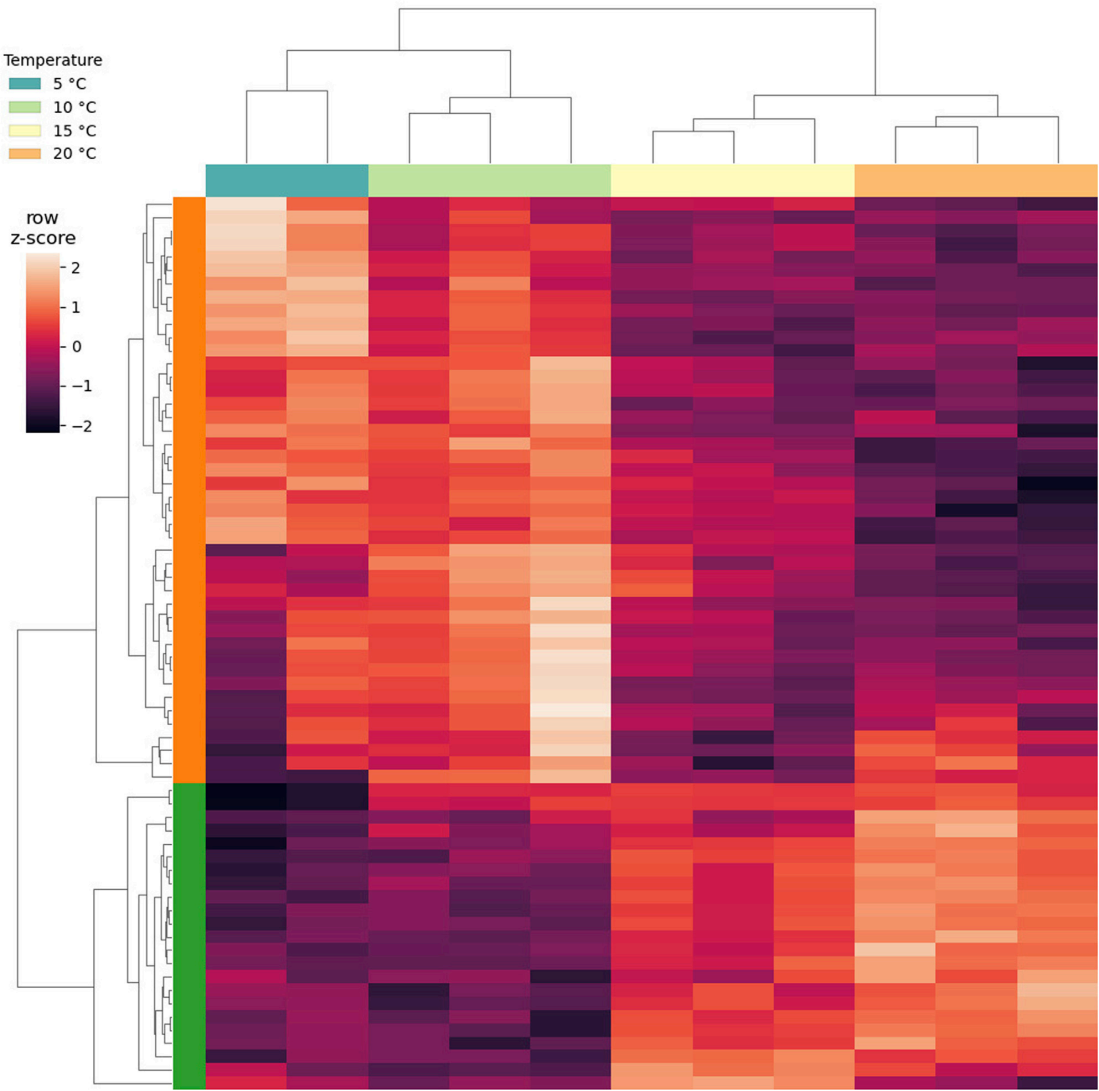
Clustered heat map of expression levels of the DEGs identified as short-term (ST) response to heat stress. Gene expression values are expressed as row-normalized, log transformed cpm. Column clusters identify exposure temperature, and each column identifies a replicate. Row clusters in green show DEGs upregulated with the increase in temperature, while row clusters in orange show DEGs downregulated with the increase in temperature.

**FIGURE 5:**
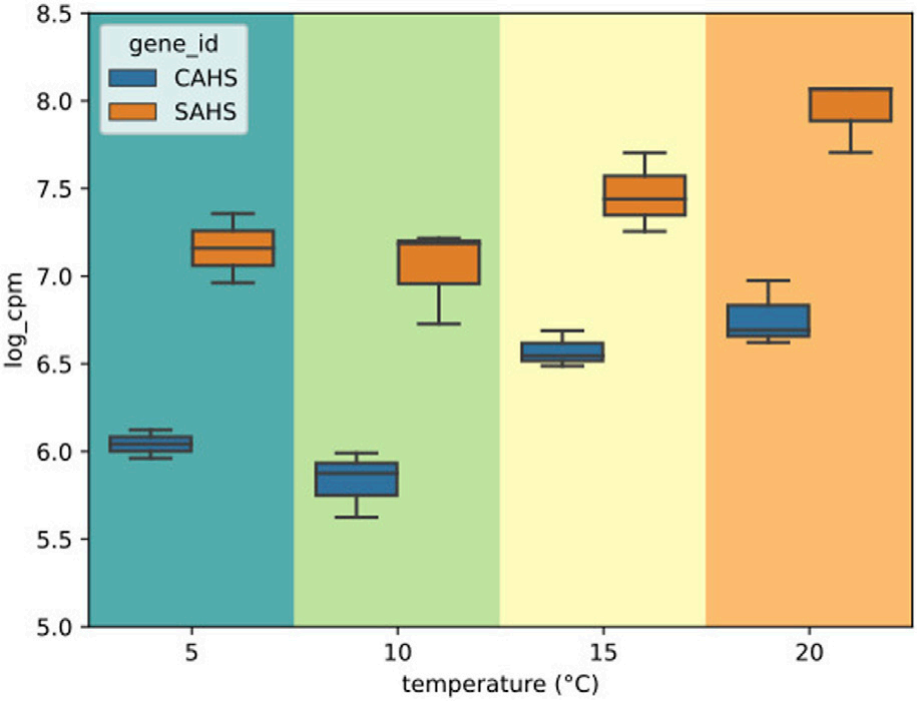
Box plots of the expression values of CAHS and SAHS genes in the short-term (ST) heat exposure experiments in log (cpm). Within each box, horizontal black lines denote median values; boxes extend from the 25th to the 75th percentile of each group’s distribution of values; vertical extending lines denote adjacent values (i.e., the most extreme values within the 1.5 interquartile range of the 25th and 75th percentile of each group).

Overall, DGE could be identified only by independently analyzing the LT and ST groups (Supplementary file S8). A total of 67 DEGs was found to be altered by short-term exposure. These DEGs showed two main temperature-dependent patterns: 23 were upregulated with the increase in temperature, and 44 were downregulated with the increase in temperature (Figure 4). The GO enrichment on the upregulated subset of DEGs suggested that processes related to mitochondria were being activated, while no significant GO term could be enriched from the downregulated genes (Supplementary file S8). In this subset, a PFAM entry could be enriched, namely, *Pupal cuticle protein C1*, which displayed an FDR of 6.74 × 10^−12^ and a O/E value of 182.79. Interestingly, genes encoding for intrinsically disordered proteins CAHS and SAHS were included in the group of DEGs that are upregulated with temperature (Figure 5). Within this group, all samples exposed to 15°C were excluded due to their high intra-replicate diversity. Nonetheless, we identified 14 DEGs that responded to the temperature increase. Specifically, two DEGs were upregulated, while the remaining 12 were downregulated. Of these, only two could be annotated based on their similarity with sequences from the *H. exemplaris* genome. The first one is OQV21646.1, which encodes for a protein containing a von Willebrand factor A domain and was found to be downregulated. The second one is OQV17502.1, which is annotated as a putative *conserved regulator of innate immunity protein 3* and was upregulated.

## Discussion

The eutardigrade *Acutuncus antarcticus* was considered an animal model to study the effects of increasing temperature due to global climate change on Antarctic ecosystems. The life cycle and the fitness of this species at different increasing temperatures were investigated to simulate the ongoing global warming. The most surprising result was the maximum life span of 686 days (about 23 months) reached by a specimen of the F_2_ generation reared at 5°C, in contrast with the maximum value of 130 days reached by a F_1_ specimen reared at 15°C (Altiero et al., 2015). A life span so long has never been recorded before in tardigrade species (Altiero et al., 2018), where the longest record of longevity was 518 days (about 17 months) for a clonal strain of the terrestrial eutardigrade *Paramacrobiotus fairbanksi* (Altiero et al., 2006), and 18 months for the marine heterotardigrade *Halobiotus crispae* (Kristensen, 1982). This result was also unexpected, even comparing it with the life cycle in rearing conditions of other extremophile organisms inhabiting Antarctica: the rotifers *Adineta grandis* and *Philodina gregaria* lived 40 and 89 days, respectively (Dartnall, 1992); the soil nematode *Scottnema lindsayae*, reared at 10°C, lived 218 days (Overhoff et al., 1993), and the terrestrial soil nematode *Plectus murrayi*, reared at 15°C, shows an annual life cycle duration (de Tomasel et al., 2013). Consistent with our results, previous studies showed that temperature has different effects on various aspects of life cycle and development of soil nematodes, especially cold temperature produces an extended adult lifespan (Moorhead et al., 2002).

*Acutuncus antarcticus* specimens reared at 5°C lived longer, molted more times, and reached sexual maturity later than those reared at 15°C. Moreover, during their life span, animals at 5°C laid more eggs that hatched after a longer time and in lower percentage, and showed a longer interval time between successive ovipositions. Other Polar invertebrates exposed to increasing temperature showed a shorter development (Yoon et al., 2023) and their eggs hatched earlier (Weydmann et al., 2015), even though their hatching success decreased (Birkemoe and Leinaas, 2000). In *A. antarcticus*, the egg hatching time also decreased when the eggs were exposed to 15°C, although their hatching percentage increased. This increased hatching success could balance the low number of laid eggs when tardigrades were reared at the highest temperature. Overall, this strategy could represent a warm climatic adaptation in response to the unpredictable Antarctic environmental conditions exhibiting temperature fluctuations even within the same day (Altiero et al., 2015).

Differences among generations of *A. antarcticus* reared at two different temperatures were evidenced in relation to some life history traits. In particular, animals belonging to the first generation lived longer than the second generation. The interval time between successive ovipositions was minimum for the animals of parental generation, and it increased for the filial generations. As a possible adaptive strategy to balance the longer interval time between ovipositions, the eggs laid by the F_2_ generation hatched earlier than other eggs. The hatching percentage was higher in eggs laid by the parental generation, and it strongly decreased in eggs laid by filial generations. The unhatched eggs could however be resting eggs, needing a stimulus such as desiccation-rehydration or freezing-thawing to hatch, as observed in other tardigrade species (Altiero et al., 2010; Møbjerg and Neves, 2021). Furthermore, the animals belonging to the first generation, reared at both tested temperatures (5°C and 15°C), showed the highest fitness. Otherwise, the fitness of the F_2_ generation at 15°C was significantly lower than the fitness of the other animal groups. These data suggest that the first generation is able to acclimate to the increasing temperature, while the second one is negatively affected. As a whole, the differences among generations in the fitness and in the life history traits, especially the ones associated with reproduction, could be due to genetic and maternal effects, and consequently to phenotypic plasticity (Marshall and Uller, 2007; Yeates et al., 2009; Fusco and Minelli, 2010; Altiero et al., 2015; Pazzaglia et al., 2021).

The first transcriptome of *A. antarcticus* exposed to increasing temperature was obtained. Upon a first analysis, expression data showed a high degree of inter sample diversity, which could not be ascribable to experimental variables. Such an effect could be the result of an unexpectedly high inter-individual diversity, which in turn could explain the unexpectedly low total number of statistically significant DEGs. The high duplication rate resulting from the BUSCO results could indeed indicate high heterozygosity, but this needs to be supported by genomic data, which are not yet available for this species. After removing the unwanted variability, a set of DEGs for both exposure times could be found. Finally, the reduced availability of high-quality genomes of tardigrade species represents a limitation to the precise annotation of transcripts in our assembled transcriptome, restricting the retrieval of information to databases such as UniProt/SwissProt and PFAM. 

When animals were exposed for 1 day to heat (short-term), 67 DEGs were found. The expression patterns of such genes showed a linearity between expression fold change and applied temperature, reinforcing the idea that the observed alterations are a transcriptional response to the temperature increase. While the difference in gene expression is small between 5°C and 10°C, the changes become evident at 15°C and even more dramatic at 20°C. The 23 upregulated genes were significantly enriched in GO terms suggesting alterations of mitochondrial activity and oxido-reductive processes. Among these, *NADH-ubiquinone oxidoreductase chain 4* and *5*, *cytochrome b*, and *cytochrome c oxidase subunits 1* and *2* are involved in the respiratory chain (Sousa et al., 2018). Since *NADH-ubiquinone oxidoreductase* is a major source of Reactive Oxygen Species (ROS) in mitochondria (Kusmann and Hirst, 2006; Esterházy et al., 2008), its upregulation, evidenced also in other tardigrade species exposed to heat stress (Neves et al., 2022), induces cellular oxidative stress. Moreover, as evidenced by Oomen et al. (2022), transcripts involved in cellular respiration are upregulated in response to heat, increasing metabolic energy requirements, oxygen consumption, and ROS, damaging complex molecules and cellular structures. The downregulation of two peroxidase (*peroxidasin* and *chorion peroxidase*) with the increasing temperature is in line with an oxidative stress scenario, since their functions are related to the protection of animals and eggs from ROS (Benoit et al., 2012; Schokraie et al., 2012).

Nudix hydrolases are widely found in prokaryotes and eukaryotes, and are involved in a variety of cellular processes such as cellular metabolism, homeostasis, and mRNA processing (Yoon et al., 2018; Yang et al., 2022). The observed upregulation of Nudix hydrolases with increasing temperature suggests a role in defense against oxidative stress, as it has already demonstrated in potato (Brouwer et al., 2021).

Disordered proteins help mediate tolerance to different abiotic stresses including freezing, osmotic stress, high temperatures, and desiccation in several organisms (Hesgrove and Boothby, 2020). Recently, three novel families of intrinsically disordered proteins (IDP) were discovered in tardigrades and revealed to contribute to a general stress response (Yamaguchi et al., 2012; Tanaka et al., 2015; Boothby et al., 2017; Hesgrove and Boothby, 2020; Yagi-Utsumi et al., 2021; Neves et al., 2022; Krakowiak et al., 2023). In this study, two intrinsically disordered protein genes, namely, Cytosolic-abundant heat soluble protein (CAHS) and secretory-abundant heat soluble protein (SAHS), were upregulated in response to heating. The aforementioned genes refer to several assembled transcripts (40 and 8 respectively) that were grouped under the same gene by best similarity and, due to the absence of a reference genome, may represent splicing isoforms and/or paralogous genes as well. As previously evidenced in *Ramazzottius varieornatus* for other IDPs (Neves et al., 2022), these obtained results confirm the role of tardigrade IDPs to cope with heat stress. The putative gene for *conserved regulator of innate immunity protein 3* was also upregulated with the increasing temperature. This gene is described in nematodes as homologous to the mammalian *C1qbp* (Alper et al., 2008), which has several functions in cell activity and a key role in the activation of the complement system, and therefore of innate immunity (Ghebrehiwet and Peerschke, 2004).

Among the 44 DEGs that were downregulated with the increase in temperature, there are genes related to the protein turnover (*cathepsin B* and UBA domain-like superfamily; Förster et al., 2012), a gene (*Multidrug resistance-associated protein 1*) involved in dauer larva regulation of the nematode *Caenorhabditis elegans* (Yabe et al., 2005), and two genes (CREB-binding protein and *Homeodomain-interacting protein kinase 2*) relevant for the control of cellular proliferation, differentiation and apoptosis (Kovács et al., 2015). The loss of function of a Homeodomain-interacting protein kinase (HPK) shortens lifespan and hastens tissue aging in *C. elegans* (Berber et al., 2016). Similarly, the downregulation of HPK gene in specimens of *A. antarcticus* exposed to increasing temperature could have reduced the life span of animals reared at 15°C compared to those reared at 5°C.

Regarding the long-term (15 days) exposure, only two DEGs out of 14 could be annotated. The gene coding for a von Willebrand factor A domain containing protein (VWA) is downregulated. The VWA plays a role in cell adhesion, extracellular matrix proteins, and integrin receptors (Whittaker and Hynes, 2002). However, the oldest known proteins containing the VWA domain are intracellular and involved in functions such as transcription, DNA repair, ribosomal and membrane transport, and the proteasome (Whittaker and Hynes, 2002). The other annotated DEG (i.e., putative *conserved regulator of innate immunity protein 3*) is upregulated in response to long-term exposure as well as short-term exposure, as described in the previous paragraph. The observed decline in transcriptomic response after a 15-day exposure indicates that the differences observed in the short term are likely due to an acclimation response. It is reasonable to hypothesize that with longer exposure durations, a return to a normal transcriptional landscape would be expected, although further data is needed to confirm this assertion.

Considering that *A. antarcticus* is a pan-Antarctic species and that the average daily pond temperatures in Victoria Land (Antarctica) range between −5 and +6°C during the summer (Cucini et al., 2022), the exposure to 15°C represents an unusual condition for this species, as well as for other Antarctic organisms. Overall, our findings indicate that the short-term heat exposure leads to significant changes in the transcriptomic landscape of *A. antarcticus*, as evidenced by the differential expression of 67 genes after 1 day of heat exposure. Notably, several genes involved in oxidative metabolism and oxidative stress response display changes in their expression patterns. Nevertheless, the gene expression alterations observed after long-term exposure are limited to 14 genes, and only the upregulation of a gene (putative *conserved regulator of innate immunity protein 3*) is evidenced both after 1 and 15 days of heat exposure.

Therefore, according to the results obtained from the life history traits and gene expression this Antarctic tardigrade species could be able to acclimate to higher temperatures over time, including possible future conditions imposed by global warming.

## Data Availability

The data presented in the study are deposited in the SRA repository under the BioProject id PRJNA851942 and are public available at the link https://www.ncbi.nlm.nih.gov/bioproject/PRJNA851942.
