# Supplementary material for: Increasing temperature-driven changes in life history traits and gene expression of an Antarctic tardigrade species
Source: Front Physiol. 2023 Sep 12;14:1258932. doi: 10.3389/fphys.2023.1258932 (PMC10520964; doi:10.3389/fphys.2023.1258932)
Supplement: Supplementary file 1 [file DataSheet4.DOCX]

# Acutuncus antarcticus life cycle analysis

# Load the libraries

library(stats) # for the glm models

library(readxl) # to load excel files

library(tidyverse) # manipulate data

library(writexl) # write excel files

library(moments) # calculate kurtosis and skewness

library(ggplot2) # plotting

library(easystats) # model result reporting

library(report) # model result reporting

## Functions to calculate 95% CI of proportion data based on n. of successes and n. of trials

binom.CI.low <- function(events, #events = outcomes

trials, #number of individuals, test, etc

alpha = 0.05){

n <- trials

x <- events

p.hat <- x/n

# Calculate upper and lower limit

upper.lim <- (p.hat + (qnorm(1-(alpha/2))^2/(2*n)) + qnorm(1-(alpha/2)) * sqrt(((p.hat*(1-p.hat))/n) + (qnorm(1-(alpha/2))^2/(4*n^2))))/(1 + (qnorm(1-(alpha/2))^2/(n)))

lower.lim <- (p.hat + (qnorm(alpha/2)^2/(2*n)) + qnorm(alpha/2) * sqrt(((p.hat*(1-p.hat))/n) + (qnorm(alpha/2)^2/(4*n^2))))/(1 + (qnorm(alpha/2)^2/(n)))

out <- c(lower.lim)

names(out) <- c("lower.CI")

return(out)

#The core code for this function is from

#stats.stackexchange.com/questions/59733/can-agresti-coull-binomial#-confidence-intervals-be-negative

#and was written by stats.stackexchange.com/users/21054/coolserdash

#for more info see

# wikipedia.org/wiki/Binomial_proportion_confidence_interval

}

binom.CI.high <- function(events, #events = outcomes

trials, #number of individuals, test, etc

alpha = 0.05){

n <- trials

x <- events

p.hat <- x/n

# Calculate upper and lower limit

upper.lim <- (p.hat + (qnorm(1-(alpha/2))^2/(2*n)) + qnorm(1-(alpha/2)) * sqrt(((p.hat*(1-p.hat))/n) + (qnorm(1-(alpha/2))^2/(4*n^2))))/(1 + (qnorm(1-(alpha/2))^2/(n)))

lower.lim <- (p.hat + (qnorm(alpha/2)^2/(2*n)) + qnorm(alpha/2) * sqrt(((p.hat*(1-p.hat))/n) + (qnorm(alpha/2)^2/(4*n^2))))/(1 + (qnorm(alpha/2)^2/(n)))

out <- c(upper.lim)

names(out) <- c("upper.CI")

return(out)

#The core code for this function is from

#stats.stackexchange.com/questions/59733/can-agresti-coull-binomial#-confidence-intervals-be-negative

#and was written by stats.stackexchange.com/users/21054/coolserdash

#for more info see

# wikipedia.org/wiki/Binomial_proportion_confidence_interval

}

###############################

# Load all the data

data_hatching_time = read_xlsx("./input_data/Hatching_time.xlsx")

data_hatching_time$generation = factor(data_hatching_time$generation, levels = c("F1","P","F2"))

data_hatching_traits = read_xlsx("./input_data/Hatching_traits.xlsx")

data_hatching_traits$generation = factor(data_hatching_traits$generation, levels = c("F1","P","F2"))

data_lh_traits = read_xlsx("./input_data/Life_history_traits.xlsx")

# Create subfolders where to store results

dir.create("./output")

dir.create("./output/summary_stats")

dir.create("./output/plots_tiff")

dir.create("./output/plots_pdf")

dir.create("./output/models_results_excel")

dir.create("./output/models_results_text")

# Descriptive statistics life traits

data_lh_traits %>% gather("trait","value",5:9) %>%

group_by(trait,temperature,generation) %>%

summarise(across(value, list(mean = ~ mean(.x, na.rm=T),

sd = ~ sd(.x, na.rm=T),

n = ~ length(.x),

quantile25 = ~ quantile(.x, prob = c(0.25), na.rm=T),

mediana_quantile50 = ~ quantile(.x, prob = c(0.50), na.rm=T), # mediana

quantile75 = ~ quantile(.x, prob = c(0.75), na.rm=T),

kurtosis = ~ moments::kurtosis(.x, na.rm=T),

skewness = ~ moments::skewness(.x, na.rm=T)))) %>%

write_xlsx("./output/summary_stats/summary_lifehistory_traits.xlsx")

data_hatching_traits %>% gather("trait","value",5:7) %>%

group_by(trait, temperature, generation) %>%

summarise(across(value, list(mean = ~ mean(.x, na.rm=T),

sd = ~ sd(.x, na.rm=T),

n = ~ length(.x),

quantile25 = ~ quantile(.x, prob = c(0.25), na.rm=T),

mediana_quantile50 = ~ quantile(.x, prob = c(0.50), na.rm=T),

quantile75 = ~ quantile(.x, prob = c(0.75), na.rm=T),

kurtosis = ~ moments::kurtosis(.x, na.rm=T),

skewness = ~ moments::skewness(.x, na.rm=T))))%>%

write_xlsx("./output/summary_stats/summary_hatching_traits.xlsx")

# Plot descriptive statistics life traits

ggplot(data_lh_traits)+

geom_boxplot(aes(x = as.factor(temperature), y = life_span, fill=generation),

col = "black", alpha = 0.5, outlier.shape = 21)+

theme_modern() + # removed text angle

theme(plot.title = element_text(hjust = 0.5),

axis.text.x = element_text(size = 10, angle = 0), # setting for x axis label

axis.text.y = element_text(size = 10, angle = 0), # setting for y axis label

legend.title = element_text(size=10))+ # change legend title size

scale_fill_material_d(palette = "ice", name = "Generation")+ # change name of legend title (with capital "G")

scale_color_material_d(palette = "ice")+

ggtitle("Life span")+ ylab("Days") + xlab("Temperature (°C)")+

scale_y_continuous(breaks = seq(from = 0,to = 700,by = 100))

ggsave("./output/plots_tiff/plot_descrittive_lifespan.tiff", width = 4.28, height = 3.54)

ggsave("./output/plots_pdf/plot_life_span.pdf", width = 4.28, height = 3.54) # Modificabile con Inkscape

ggplot(data_lh_traits)+

geom_boxplot(aes(x = as.factor(temperature), y = ovip_number, fill=generation),

col = "black", alpha = 0.5, outlier.shape = 21)+

theme_modern() +

theme(plot.title = element_text(hjust = 0.5),

axis.text.x = element_text(size = 10, angle = 0), # setting for x axis label

axis.text.y = element_text(size = 10, angle = 0), # setting for y axis label

legend.title = element_text(size=10))+ # change legend title size

scale_fill_material_d(palette = "ice", name = "Generation")+ # change name of legend title (with capital "G")

scale_color_material_d(palette = "ice")+

ggtitle("Oviposition number per life span") + ylab("Oviposition number") + xlab("Temperature (°C)")+

scale_y_continuous(breaks = seq(from = 0,to = 50,by = 5))

ggsave("./output/plots_tiff/plot_descrittive_oviposition number.tiff", width = 4.28, height = 3.54)

ggsave("./output/plots_pdf/plot_ovip_number.pdf", width = 4.28, height = 3.54) # Modificabile con Inkscape

ggplot(data_lh_traits)+

geom_boxplot(aes(x = as.factor(temperature), y = age_first_ovip, fill=generation),

col = "black", alpha = 0.5, outlier.shape = 21)+

theme_modern() +

theme(plot.title = element_text(hjust = 0.5),

axis.text.x = element_text(size = 10, angle = 0), # setting for x axis label

axis.text.y = element_text(size = 10, angle = 0), # setting for y axis label

legend.title = element_text(size=10))+ # change legend title size

scale_fill_material_d(palette = "ice", name = "Generation")+ # change name of legend title (with capital "G")

scale_color_material_d(palette = "ice")+

ggtitle("Age at first oviposition") + ylab("Days") + xlab("Temperature (°C)") +

scale_y_continuous(breaks = seq(from = 0,to = 70,by = 5))

ggsave("./output/plots_tiff/plot_descrittive_first_oviposition.tiff", width = 4.28, height = 3.54)

ggsave("./output/plots_pdf/plot_first_ovip.pdf", width = 4.28, height = 3.54) # Modificabile con Inkscape

ggplot(data_lh_traits)+

geom_boxplot(aes(x = as.factor(temperature), y = total_eggs, fill=generation),

col = "black", alpha = 0.5, outlier.shape = 21)+

theme_modern() +

theme(plot.title = element_text(hjust = 0.5),

axis.text.x = element_text(size = 10, angle = 0), # setting for x axis label

axis.text.y = element_text(size = 10, angle = 0), # setting for y axis label

legend.title = element_text(size=10))+ # change legend title size

scale_fill_material_d(palette = "ice", name = "Generation")+ # change name of legend title (with capital "G")

scale_color_material_d(palette = "ice")+

ggtitle("Fecundity") + ylab("Egg number per female per life span") + xlab("Temperature (°C)") +

scale_y_continuous(breaks = seq(from = 0,to = 100,by = 10))

ggsave("./output/plots_tiff/plot_descrittive_fecundity.tiff", width = 4.28, height = 3.54)

ggsave("./output/plots_pdf/plot_fecundity.pdf", width = 4.28, height = 3.54) # Modificabile con Inkscape

ggplot(data_lh_traits)+

geom_boxplot(aes(x = as.factor(temperature), y = molt_number, fill=generation),

col = "black", alpha = 0.5, outlier.shape = 21)+

theme_modern() +

theme(plot.title = element_text(hjust = 0.5),

axis.text.x = element_text(size = 10, angle = 0), # setting for x axis label

axis.text.y = element_text(size = 10, angle = 0), # setting for y axis label

legend.title = element_text(size=10))+ # change legend title size

scale_fill_material_d(palette = "ice", name = "Generation")+ # change name of legend title (with capital "G")

scale_color_material_d(palette = "ice")+

ggtitle("Number of molts") + ylab("Number of molts") + xlab("Temperature (°C)") +

scale_y_continuous(breaks = seq(from = 0,to = 45,by = 5))

ggsave("./output/plots_tiff/plot_descrittive_molts.tiff", width = 4.28, height = 3.54)

ggsave("./output/plots_pdf/plot_molts.pdf", width = 4.28, height = 3.54) # Modificabile con Inkscape

data_hatching_traits$generation = factor(data_hatching_traits$generation, levels = c("P","F1","F2"))

ggplot(data_hatching_traits)+

geom_boxplot(aes(x = as.factor(temperature), y = time_prev_ovip, fill=generation),

col = "black", alpha = 0.5, outlier.shape = 21)+

theme_modern() +

theme(plot.title = element_text(hjust = 0.5),

axis.text.x = element_text(size = 10, angle = 0), # setting for x axis label

axis.text.y = element_text(size = 10, angle = 0), # setting for y axis label

legend.title = element_text(size=10))+ # change legend title size

scale_fill_material_d(palette = "ice", name = "Generation")+ # change name of legend title (with capital "G")

scale_color_material_d(palette = "ice")+

ggtitle("Time interval among oviposition") + ylab("Days") + xlab("Temperature (°C)") +

scale_y_continuous(breaks = seq(from = 0,to = 60,by = 5))

ggsave("./output/plots_tiff/plot_descrittive_time_interval.tiff", width = 4.28, height = 3.54)

ggsave("./output/plots_pdf/plot_time_interval.pdf", width = 4.28, height = 3.54) # Modificabile con Inkscape

# Descriptives hatching percentage

data_hatching_time %>%

group_by(temperature,generation)%>%

summarise(hatching_mean = mean(hatching, na.rm=T),

hatched = sum(hatching, na.rm=T),

n = length(hatching)) %>% as.data.frame() %>%

mutate(low95CI = binom.CI.low(as.numeric(hatched),as.numeric(n)),

high95CI = binom.CI.high(as.numeric(hatched),as.numeric(n)))%>%

write_xlsx("./output/summary_stats/summary_hatching_percentage.xlsx")

# GPlot hatching percentage

data_hatching_time %>%

group_by(temperature,generation)%>%

summarise(hatching_mean = mean(hatching, na.rm=T),

hatched = sum(hatching, na.rm=T),

n = length(hatching)) %>% ungroup() %>%

mutate(low95CI = binom.CI.low(as.numeric(hatched),as.numeric(n)),

high95CI = binom.CI.high(as.numeric(hatched),as.numeric(n))) %>%

mutate(generation = fct_relevel(generation, c("P","F1","F2"))) %>%

ggplot()+

geom_linerange(aes(x = as.factor(temperature), group=generation, ymin = low95CI, ymax = high95CI),

position = position_dodge(width = 0.5), size=0.55, alpha=1, show.legend =F, col="black")+

geom_point(aes(x = as.factor(temperature), fill=generation, y=hatching_mean),

position = position_dodge(width = 0.5), size=4.5, pch=21, alpha = 0.65)+

theme_modern() + # removed text angle

theme(plot.title = element_text(hjust = 0.5),

axis.text.x = element_text(size = 10, angle = 0), # setting for x axis label

axis.text.y = element_text(size = 10, angle = 0), # setting for y axis label

legend.title = element_text(size=10))+ # change legend title size

scale_fill_material_d(palette = "ice", name = "Generation")+ # change name of legend title (with capital "G")

scale_color_material_d(palette = "ice")+

ggtitle("Hatching percentage")+ ylab("Hatching percentage (%)") + xlab("Temperature (°C)")+

scale_y_continuous(breaks = seq(from = 0,to = 1,by = 0.1), limits = c(0,1),

labels = seq(from = 0,to = 100,by = 10))

ggsave("./output/plots_tiff/plot_hatching_perc_eggs.tiff", width = 4.28, height = 3.54)

ggsave("./output/plots_pdf/plot_hatching_perc_eggs.pdf", width = 4.28, height = 3.54) # Modificabile con Inkscape

## Descriptives hatching time

data_hatching_time %>%

group_by(temperature,generation)%>%

summarise(across(hatching_time, list(mean = ~ mean(.x, na.rm=T),

sd = ~ sd(.x, na.rm=T),

n = ~ length(.x),

quantile25 = ~ quantile(.x, prob = c(0.25), na.rm=T),

quantile50 = ~ quantile(.x, prob = c(0.50), na.rm=T), # mediana

quantile75 = ~ quantile(.x, prob = c(0.75), na.rm=T),

kurtosis = ~ moments::kurtosis(.x, na.rm=T),

skewness = ~ moments::skewness(.x, na.rm=T)))) %>%

write_xlsx("./output/summary_stats/summary_hatching_time.xlsx")

## Plot hatching time

data_hatching_time %>% mutate(generation = fct_relevel(generation, c("P","F1","F2"))) %>%

ggplot()+

geom_boxplot(aes(x = as.factor(temperature), y = hatching_time, fill=generation),

col = "black", alpha = 0.5, outlier.shape = 21)+ # color outlier points

theme_modern() + # removed text angle

theme(plot.title = element_text(hjust = 0.5),

axis.text.x = element_text(size = 10, angle = 0), # setting for x axis label

axis.text.y = element_text(size = 10, angle = 0), # setting for y axis label

legend.title = element_text(size=10))+ # change legend title size

scale_fill_material_d(palette = "ice", name = "Generation")+ # change name of legend title (with capital "G")

scale_color_material_d(palette = "ice")+

ggtitle("Hatching time")+ ylab("Days") + xlab("Temperature (°C)")+

scale_y_continuous(breaks = seq(from = 0,to = 50,by = 5)) # nell'asse delle y metti i numeri da 0 a 600, ogni 100

ggsave("./output/plots_tiff/plot_hatching_time.tiff", width = 4.28, height = 3.54)

ggsave("./output/plots_pdf/plot_hatching_time.pdf", width = 4.28, height = 3.54) # Modificabile con Inkscape

#### Descriptives and plot for # eggs per clutch

clutch_count_tab_max = data_hatching_traits %>% mutate(check = 1) %>% mutate(generation = fct_relevel(generation, c("P", "F1", "F2"))) %>%

group_by(generation, temperature) %>%

summarise(across(check, list(n = ~ length(.x)))) %>% mutate(check_max = check_n)

clutch_count_tab_max = clutch_count_tab_max[,c(1,2,4)]

clutch_count_tab = data_hatching_traits %>% mutate(check = 1) %>% mutate(generation = fct_relevel(generation, c("P", "F1", "F2"))) %>%

group_by(generation, temperature,eggs_clutch) %>%

summarise(across(check, list(n = ~ length(.x)))) %>% merge(clutch_count_tab_max) %>%

mutate(prop_clutch_size = check_n/check_max)

ggplot(clutch_count_tab)+

geom_point(aes(x = as.factor(temperature), y = eggs_clutch, fill=generation, size = prop_clutch_size),

pch=21, position = position_dodge(width = 0.75), alpha=0.35, col = "black")+

theme_modern(axis.text.angle = 45) +

theme(plot.title = element_text(hjust = 0.5))+

scale_fill_material_d(palette = "ice")+

scale_color_material_d(palette = "ice")+

scale_size_continuous(range = c(0.1,6.5))+

ggtitle("Fertility")+ ylab("Egg number per clutch") + xlab("Temperature (°C)")+

scale_y_continuous(breaks=seq(1,7,by=1))

ggsave("./output/plots_tiff/plot_fertility.tiff", width = 4.28, height = 3.54)

ggsave("./output/plots_pdf/plot_fertility.pdf", width = 4.28, height = 3.54) # Modificabile con Inkscape

### RUN GLMs models

# Relevel the data factors

data_lh_traits$generation = factor(data_lh_traits$generation, levels = c("F1","F2"))

data_lh_traits$temperature = factor(data_lh_traits$temperature, levels = c("5","15"))

data_hatching_traits$generation = factor(data_hatching_traits$generation, levels = c("F1","P","F2"))

data_hatching_traits$temperature = factor(data_hatching_traits$temperature, levels = c("5","15"))

data_hatching_time$generation = factor(data_hatching_time$generation, levels = c("F1","P","F2"))

data_hatching_time$temperature = factor(data_hatching_time$temperature, levels = c("5","15"))

# LIFESPAN

mod_lifespan = glm(life_span ~ generation + temperature + generation:temperature,

data = data_lh_traits,

family = poisson)

cat(report::report(mod_lifespan), file = "./output/models_results_text/lifespan.txt")

parameters::parameters(mod_lifespan)%>%

write_xlsx("./output/models_results_excel/lifespan.xlsx")

# NUMBER OF OVIPOSITIONS

mod_ovip_number = glm(ovip_number ~ generation + temperature + generation:temperature,

data = data_lh_traits,

family = poisson)

cat(report::report(mod_ovip_number), file = "./output/models_results_text/oviposition_number.txt")

parameters::parameters(mod_ovip_number)%>%

write_xlsx("./output/models_results_excel/oviposition_number.xlsx")

# TOTAL EGGS LAID

mod_total_eggs = glm(total_eggs ~ generation + temperature + generation:temperature,

data = data_lh_traits,

family = poisson)

cat(report::report(mod_total_eggs), file = "./output/models_results_text/total_eggs.txt")

parameters::parameters(mod_total_eggs)%>%

write_xlsx("./output/models_results_excel/total_eggs.xlsx")

# AGE OF FIRST OVIPOSITION

mod_age_first_ovip = glm(age_first_ovip ~ generation + temperature + generation:temperature,

data = data_lh_traits,

family = poisson)

cat(report::report(mod_age_first_ovip), file = "./output/models_results_text/age_first_ovip.txt")

parameters::parameters(mod_age_first_ovip)%>%

write_xlsx("./output/models_results_excel/age_first_ovip.xlsx")

# NUMBER OF MOLTS

mod_molt_number = glm(molt_number ~ generation + temperature + generation:temperature,

data = data_lh_traits,

family = poisson)

cat(report::report(mod_molt_number), file = "./output/models_results_text/molt_number.txt")

parameters::parameters(mod_molt_number)%>%

write_xlsx("./output/models_results_excel/molt_number.xlsx")

# TIME BETWEEN OVIPOSITIONS

mod_time_prev_ovip = glm(time_prev_ovip ~ generation + temperature + generation:temperature,

data = data_hatching_traits,

family = poisson)

cat(report::report(mod_time_prev_ovip), file = "./output/models_results_text/time_between_ovipositions.txt")

parameters::parameters(mod_time_prev_ovip)%>%

write_xlsx("./output/models_results_excel/time_between_ovipositions.xlsx")

# CLUTCH SIZE

mod_eggs_clutch_order = glm(eggs_clutch ~ order_ovip + temperature + generation + generation:temperature,

data = subset(data_hatching_traits, generation !="P"),

family = poisson)

cat(report::report(mod_eggs_clutch_order), file = "./output/models_results_text/clutch_size.txt")

parameters::parameters(mod_eggs_clutch_order)%>%

write_xlsx("./output/models_results_excel/clutch_size.xlsx")

# HATCHING PROPORTION

mod_hatching_perc = glm(hatching ~ generation + temperature + generation:temperature + scale(clutch_size),

data = data_hatching_time,

family = binomial)

cat(report::report(mod_hatching_perc), file = "./output/models_results_text/hatching_proportion.txt")

parameters::parameters(mod_hatching_perc)%>%

write_xlsx("./output/models_results_excel/hatching_proportion.xlsx")

# HATCHING TIME

mod_hatch_time = glm(hatching_time ~ generation + temperature + generation:temperature + scale(clutch_size),

data = data_hatching_time,

family = poisson)

cat(report::report(mod_hatch_time), file = "./output/models_results_text/hatching_time.txt")

parameters::parameters(mod_hatch_time)%>%

write_xlsx("./output/models_results_excel/hatching_time.xlsx")
