# Supplementary material for: Increasing temperature-driven changes in life history traits and gene expression of an Antarctic tardigrade species
Source: Front Physiol. 2023 Sep 12;14:1258932. doi: 10.3389/fphys.2023.1258932 (PMC10520964; doi:10.3389/fphys.2023.1258932)
Supplement: Supplementary file 2 [file Table1.DOCX]

**Tab. S1.** Reports of generalized linear models (GLM) generated with the R package.

| **Response** | **Formula** | **Family** |
| --- | --- | --- |
| life span | ~ generation + temperature + generation : temperature | Poisson |
| number of molts | ~ generation + temperature + generation : temperature | Poisson |
| age at first and last oviposition | ~ generation + temperature + generation : temperature | Poisson |
| number of ovipositions per life span | ~ generation + temperature + generation : temperature | Poisson |
| number of laid eggs per life span (fecundity) | ~ generation + temperature + generation : temperature | Poisson |
| number of eggs per clutch (fertility) | ~ order of oviposition + temperature + generation +     generation : temperature | Poisson |
| interval of time between ovipositions | ~ generation + temperature + generation : temperature | Poisson |
| egg hatching time | ~ generation + temperature + generation : temperature +     scale (number of eggs per clutch) | Poisson |
| egg hatching percentage | ~ generation + temperature + generation : temperature +     scale (number of eggs per clutch) | Binomial |

~ predicted by; + additive effect; : interaction
