# Supplementary material for: Increasing temperature-driven changes in life history traits and gene expression of an Antarctic tardigrade species
Source: Front Physiol. 2023 Sep 12;14:1258932. doi: 10.3389/fphys.2023.1258932 (PMC10520964; doi:10.3389/fphys.2023.1258932)
Supplement: Supplementary file 4 [file DataSheet5.DOCX]

**Supplementary material S2 “Significances of GLM”**

*GLM results: life span*

The model’s explanatory power is substantial (Nagelkerke’s R_2_ = 1.00). The model’s intercept, corresponding to generation = F_1_, is at 6.29 (95% CI [6.27, 6.31], *p <* 0.001). Within this model:

- the effect of generation [F_2_] is statistically significant and negative (beta = -0.13, 95% CI [-0.15, -0.10], *p <* 0.001; Std. beta = -0.13, 95% CI [-0.15, -0.10]);

- the effect of temperature [15 °C] is statistically significant and negative (beta = -1.81, 95% CI [-1.86, -1.76], *p <* 0.001; Std. beta = -1.81, 95% CI [-1.86, -1.76]);

- the interaction effect of temperature [15 °C] on generation [F_2_] is statistically significant and negative (beta = -0.46, 95% CI [-0.53, -0.39], *p <* 0.001; Std. beta = -0.46, 95% CI [-0.53, -0.39]).

*GLM results: number of molts*

The model’s explanatory power is substantial (Nagelkerke’s R_2_ = 1.00). The model’s intercept, corresponding to generation = F_1_, is at 3.44 (95% CI [3.35, 3.52], *p <* 0.001). Within this model:

- the effect of generation [F_2_] is statistically significant and negative (beta = -0.19, 95% CI [-0.30, -0.08], *p <* 0.001; Std. beta = -0.19, 95% CI [-0.30, -0.08]);

- the effect of temperature [15 °C] is statistically significant and negative (beta = -1.45, 95% CI [-1.62, -1.27], *p <* 0.001; Std. beta = -1.45, 95% CI [-1.62, -1.27]);

- the interaction effect of temperature [15 °C] on generation [F_2_] is statistically significant and negative (beta = -0.43, 95% CI [-0.69, -0.18], *p <* 0.001; Std. beta = -0.43, 95% CI [-0.69, -0.18]).

*GLM results: age at first oviposition*

The model’s explanatory power is substantial (Nagelkerke’s R_2_ = 0.96). The model’s intercept, corresponding to generation = F_1_, is at 3.52 (95% CI [3.47, 3.57], *p <* 0.001). Within this model:

- the effect of generation [F_2_] is statistically non-significant and positive (beta = 4.42e-03, 95% CI [-0.08, 0.09], *p* = 0.915; Std. beta = 4.42e-03, 95% CI [-0.08, 0.09]);

- the effect of temperature [15 °C] is statistically significant and negative (beta = -0.68, 95% CI [-0.80, -0.57], *p <* 0.001; Std. beta = -0.68, 95% CI [-0.80, -0.57]);

- the interaction effect of temperature [15 °C] on generation [F_2_] is statistically non-significant and negative (beta = -0.01, 95% CI [-0.17, 0.14], *p* = 0.864; Std. beta = -0.01, 95% CI [-0.17, 0.14]).

*GLM results: number of ovipositions per life span*

The model’s explanatory power is substantial (Nagelkerke’s R_2_ = 1.00). The model’s intercept, corresponding to generation = F_1_, is at 3.39 (95% CI [3.31, 3.48], *p <* 0.001). Within this model:

- the effect of generation [F_2_] is statistically significant and negative (beta = -0.19, 95% CI [-0.31, -0.08], *p* = 0.001; Std. beta = -0.19, 95% CI [-0.31, -0.08]);

- the effect of temperature [15 °C] is statistically significant and negative (beta = -1.54, 95% CI [-1.73, -1.36], *p <* 0.001; Std. beta = -1.54, 95% CI [-1.73, -1.36]);

- the interaction effect of temperature [15 °C] on generation [F_2_] is statistically significant and negative (beta = -0.55, 95% CI [-0.84, -0.26], *p <* 0.001; Std. beta = -0.55, 95% CI [-0.84, -0.26]).

*GLM results: interval of time between ovipositions*

The model’s explanatory power is substantial (Nagelkerke’s R_2_ = 0.78). The model’s intercept, corresponding to generation = F_1_, is at 2.79 (95% CI [2.77, 2.81], *p <* 0.001). Within this model:

- the effect of generation [P] is statistically significant and negative (beta = -0.23, 95% CI [-0.29, -0.16], *p <* 0.001; Std. beta = -0.23, 95% CI [-0.29, -0.16]);

- the effect of generation [F_2_] is statistically significant and positive (beta = 0.03, 95% CI [4.75e-03, 0.06], *p* = 0.022; Std. beta = 0.03, 95% CI [4.75e-03, 0.06]);

- the effect of temperature [15 °C] is statistically significant and negative (beta = -0.94, 95% CI [-1.00, -0.88], *p <* 0.001; Std. beta = -0.94, 95% CI [-1.00, -0.88]);

- the interaction effect of temperature [15 °C] on generation [P] is statistically significant and positive (beta = 0.28, 95% CI [0.13, 0.42], *p <* 0.001; Std. beta = 0.28, 95% CI [0.13, 0.42]);

- the interaction effect of temperature [15 °C] on generation [F_2_] is statistically non-significant and positive (beta = 0.06, 95% CI [-0.05, 0.18], *p* = 0.262; Std. beta = 0.06, 95% CI [-0.05, 0.18]).

*GLM results: fecundity*

The model’s explanatory power is substantial (Nagelkerke’s R_2_ = 1.00). The model’s intercept, corresponding to generation = F_1_, is at 4.37 (95% CI [4.31, 4.42], *p <* 0.001). Within this model:

- the effect of generation [F_2_] is statistically significant and negative (beta = -0.23, 95% CI [-0.30, -0.16], *p <* 0.001; Std. beta = -0.23, 95% CI [-0.30, -0.16]);

- the effect of temperature [15 °C] is statistically significant and negative (beta = -1.80, 95% CI [-1.93, -1.68], *p <* 0.001; Std. beta = -1.80, 95% CI [-1.93, -1.68]);

- the interaction effect of temperature [15 °C] on generation [F_2_] is statistically significant and negative (beta = -0.68, 95% CI [-0.89, -0.47], *p <* 0.001; Std. beta = -0.68, 95% CI [-0.89, -0.47]).

*GLM results: fertility*

The model’s explanatory power is substantial (Nagelkerke’s R_2_ = 0.28). The model’s intercept, corresponding to the order of oviposition = 0, is at 0.64 (95% CI [0.56, 0.72], *p <* 0.001). Within this model: - the effect of the order of oviposition is statistically significant and positive (beta = 0.02, 95% CI [0.02, 0.02], *p <* 0.001; Std. beta = 0.18, 95% CI [0.14, 0.21]);

- the effect of temperature [15 °C] is statistically non-significant and negative (beta = -0.10, 95% CI [-0.21, 0.02], *p* = 0.102; Std. beta = -0.10, 95% CI [-0.21, 0.02]);

- the effect of generation [F_2_] is statistically non-significant and positive (beta = 0.01, 95% CI [-0.06, 0.08], *p* = 0.774; Std. beta = 0.01, 95% CI [-0.06, 0.08]);

- the interaction effect of generation [F_2_] on temperature [15 °C] is statistically non-significant and negative (beta = -0.06, 95% CI [-0.25, 0.13], p = 0.554; Std. beta = -0.06, 95% CI [-0.25, 0.13]).

*GLM results: egg hatching time*

The model’s explanatory power is substantial (Nagelkerke’s R_2_ = 0.94). The model’s intercept, corresponding to generation = F_1_, is at 3.08 (95% CI [3.06, 3.10], *p <* 0.001). Within this model:

- the effect of generation [P] is statistically significant and negative (beta = -0.34, 95% CI [-0.38, -0.29], *p <* 0.001; Std. beta = -0.34, 95% CI [-0.38, -0.29]);

- the effect of generation [F_2_] is statistically non-significant and positive (beta = 9.18e-03, 95% CI [-0.02, 0.04], *p* = 0.528; Std. beta = 9.18e-03, 95% CI [-0.02, 0.04]);

- the effect of temperature [15 °C] is statistically significant and negative (beta = -0.83, 95% CI [-0.88, -0.79], *p <* 0.001; Std. beta = -0.83, 95% CI [-0.88, -0.79]);

- the effect of number of eggs per clutch is statistically significant and positive (beta = 0.08, 95% CI [0.07, 0.10], *p <* 0.001; Std. beta = 0.09, 95% CI [0.08, 0.11]);

- the interaction effect of temperature [15] on generation [P] is statistically significant and positive (beta = 0.32, 95% CI [0.23, 0.41], p < .001; Std. beta = 0.32, 95% CI [0.23, 0.41])

- The interaction effect of temperature [15 °C] on generation [F_2_] is statistically significant and negative (beta = -0.15, 95% CI [-0.27, -0.04], *p* = 0.007; Std. beta = -0.15, 95% CI [-0.27, -0.04]).

*GLM results: egg hatching percentage*

The model’s explanatory power is weak (Tjur’s R_2_ = 0.11). The model’s intercept, corresponding to generation = F*1*, is at -0.87 (95% CI [-0.98, -0.76], *p <* 0.001). Within this model:

- the effect of generation [P] is statistically significant and positive (beta = 1.48, 95% CI [1.19, 1.77], *p <* 0.001; Std. beta = 1.48, 95% CI [1.19, 1.77]);

- the effect of generation [F_2_] is statistically significant and negative (beta = -0.24, 95% CI [-0.40, -0.08], *p* = 0.003; Std. beta = -0.24, 95% CI [-0.40, -0.08]);

- the effect of temperature [15 °C] is statistically significant and positive (beta = 1.34, 95% CI [1.11, 1.56], *p <* 0.001; Std. beta = 1.34, 95% CI [1.11, 1.56]);

- The effect of number of eggs per clutch is statistically significant and positive (beta = 0.13, 95% CI [0.06, 0.21], *p <* 0.001; Std. beta = 0.13, 95% CI [0.06, 0.21]);

- the interaction effect of temperature [15] on generation [P] is statistically significant and negative (beta = -0.63, 95% CI [-1.17, -0.06], p = 0.028; Std. beta = -0.63, 95% CI [-1.17, -0.06])

- The interaction effect of temperature [15 °C] on generation [F_2_] is statistically significant and negative (beta = -0.97, 95% CI [-1.39, -0.56], *p <* 0.001; Std. beta = -0.97, 95% CI [-1.39, -0.56]).
