# Supplementary material for: Increasing temperature-driven changes in life history traits and gene expression of an Antarctic tardigrade species
Source: Front Physiol. 2023 Sep 12;14:1258932. doi: 10.3389/fphys.2023.1258932 (PMC10520964; doi:10.3389/fphys.2023.1258932)
Supplement: Supplementary file 6 [file Table2.DOCX]

**Tab. S2.** Life history traits of the three generations (P, F_1_, F_2_) of *Acutuncus antarcticus* reared at the temperature of 5 °C.

| Life history traits | Generation |  |  |
| --- | --- | --- | --- |
|  | *P* | *F_1_* | *F_2_* |
| Life span (days) |  |  |  |
| Sample size |  | 40 | 31 |
| Mean ± Standard Deviation (SD) |  | 541.3 ± 60.7 | 477.4 ± 165.5 |
| Median (percentiles 25; 75) |  | 544.5 (515.0; 579.5) | 525.5 (469.3; 558.8) |
| Skewness |  | 5.083 | 3.923 |
| Kurtosis |  | -1.137 | -1.425 |
| Number of molts |  |  |  |
| Sample size |  | 40 | 31 |
| Mean ± SD |  | 31.1 ± 3.4 | 25.6 ± 8.7 |
| Median (percentiles 25; 75) |  | 31.0 (30.0; 33.0) | 28.0 (26.0; 31.0) |
| Skewness |  | 5.531 | 4.022 |
| Kurtosis |  | -1.322 | -1.483 |
| Age at first oviposition |  |  |  |
| Sample size |  | 40 | 31 |
| Mean ± SD |  | 33.9 ± 8.0 | 34.0 ± 5.2 |
| Median (percentiles 25; 75) |  | 30.0 (27.8; 38.3) | 33.5 (29.5; 36.0) |
| Skewness |  | 4.627 | 2.958 |
| Kurtosis |  | 1.407 | 0.639 |
| Oviposition number per life span |  |  |  |
| Sample size |  | 40 | 31 |
| Mean ± SD |  | 29.8 ± 3.5 | 24.6 ± 9.1 |
| Median (percentiles 25; 75) |  | 30.0 (27.5; 32.0) | 28.0 (25.0; 30.0) |
| Skewness |  | 4.550 | 4.056 |
| Kurtosis |  | -1.188 | -1.534 |
| Interval of time between ovipositions (days) |  |  |  |
| Sample size | 81 | 605 | 657 |
| Mean ± SD | 13.0 ± 3.8 | 16.2 ± 4.6 | 16.8 ± 5.0 |
| Median (percentiles 25; 75) | 13.0 (10.0; 14.0) | 15.0 (14.0; 18.0) | 17.0 (14.0; 19.0) |
| Skewness | 3.331 | 17.731 | 10.713 |
| Kurtosis | 0.466 | 2.668 | 1.749 |
| Number of eggs per female per life span (fecundity) |  |  |  |
| Sample size |  | 40 | 31 |
| Mean ± SD |  | 78.8 ± 12.6 | 62.8 ± 25.7 |
| Median (percentiles 25; 75) |  | 82.5 (73.8; 88.0) | 74.5 (66.8; 77.0) |
| Skewness |  | 2.823 | 3.453 |
| Kurtosis |  | -0.912 | -1.467 |
| Number of eggs per clutch (fertility) |  |  |  |
| Sample size | 81 | 605 | 657 |
| Mean ± SD | 3.1 ± 1.7 | 2.5 ± 1.0 | 2.6 ± 1.0 |
| Median (percentiles 25; 75) | 3.0 (2.0; 4.0) | 3.0 (2.0; 3.0) | 3.0 (2.0; 3.0) |
| Skewness | 2.343 | 2.507 | 2.619 |
| Kurtosis | 0.586 | 0.255 | 0.212 |
| Hatching time (days) |  |  |  |
| Sample size | 255 | 1545 | 1678 |
| Mean ± SD | 17.5 ± 2.6 | 21.9 ± 4.8 | 22.4 ± 5.4 |
| Median (percentiles 25; 75) | 17.0 (16.0; 19.0) | 21.0 (25.0; 18.0) | 22.0 (25.0; 19.0) |
| Skewness | 5.890 | 2.679 | 0.432 |
| Kurtosis | 0.337 | 2.979 | 0.151 |
| Hatching percentage |  |  |  |
| Sample size | 255 | 1545 | 1678 |
| Number of hatched eggs | 173 | 453 | 410 |
| Mean (95% C.I.) | 67.8 (61.9 – 73.3) | 29.8 (27.1 – 31.6) | 25.0 (22.4 – 26.5) |
