# Supplementary material for: Increasing temperature-driven changes in life history traits and gene expression of an Antarctic tardigrade species
Source: Front Physiol. 2023 Sep 12;14:1258932. doi: 10.3389/fphys.2023.1258932 (PMC10520964; doi:10.3389/fphys.2023.1258932)
Supplement: Supplementary file 8 [file DataSheet7.ZIP › Supplementary file S7.html]

MultiQC Report


# Toggle navigation v0.9 (b'0d7f54d')

- General Stats
- FastQC
  - Sequence Quality Histograms
  - Per Sequence Quality Scores
  - Per Base Sequence Content
  - Per Sequence GC Content
  - Per Base N Content
  - Sequence Length Distribution
  - Sequence Duplication Levels
  - Overrepresented sequences
  - Adapter Content

Toolbox

### MultiQC Toolbox

#### Apply Highlight Samples

+

Regex mode off
help
 Clear

#### Apply Rename Samples

+

Click here for bulk input.

Paste two columns of a tab-delimited table here (eg. from Excel).

First column should be the old name, second column the new name.

Add

Regex mode off
help
 Clear

#### Apply Show / Hide Samples

Hide matching samples

Show only matching samples

+

Regex mode off
help
 Clear

#### Export Plots

- Images
- Data

px

px

Aspect ratio

PNG
JPEG
SVG

Plot scaling

X

Download the raw data used to create the plots in this report below:

Format:

Tab-separated
Comma-separated
JSON

Note that additional data was saved in `multiqc_data` when this report was generated.

---

##### Choose Plots

 All
 None

---

   Download Plot Images

If you use plots from MultiQC in a publication or presentation, please cite:

> **MultiQC: Summarize analysis results for multiple tools and samples in a single report**  
> *Philip Ewels, Måns Magnusson, Sverker Lundin and Max Käller*  
> Bioinformatics (2016)  
> doi: 10.1093/bioinformatics/btw354  
> PMID: 27312411

#### Save Settings

You can save the toolbox settings for this report to the browser.

 Save


---

#### Load Settings

Choose a saved report profile from the dropdown box below:

[ select ]

Load
 Delete

#### About MultiQC

This report was generated using MultiQC, version 0.9 (b'0d7f54d')

You can see a YouTube video describing how to use MultiQC reports here:
https://youtu.be/qPbIlO\_KWN0

For more information about MultiQC, including other videos and
extensive documentation, please visit http://multiqc.info

You can report bugs, suggest improvements and find the source code for MultiQC on GitHub:
https://github.com/ewels/MultiQC

MultiQC is published in Bioinformatics:

> **MultiQC: Summarize analysis results for multiple tools and samples in a single report**  
> *Philip Ewels, Måns Magnusson, Sverker Lundin and Max Käller*  
> Bioinformatics (2016)  
> doi: 10.1093/bioinformatics/btw354  
> PMID: 27312411

# 

A modular tool to aggregate results from bioinformatics analyses across many samples into a single report.

Report generated on 2019-12-11, 14:12 based on data in:
`/mnt/DATA/Shared/Whole/Acutuncus_antarticus/FastQC`

---

×
don't show again

**Welcome!** Not sure where to start?  
Watch a tutorial video
  *(6:06)*

## General Statistics

 Copy table

 Configure Columns

 Sort by highlight

 Plot
Showing 54/54 rows and 4/5 columns.

| Sample Name | % Dups | % GC | Length | % Failed | M Seqs |
| --- | --- | --- | --- | --- | --- |
| 10-BT-7\_S11\_L001\_R1\_001 | 75.5% | 49% | 142 | 27% | 197.8 |
| 10-BT-7\_S11\_L001\_R2\_001 | 78.7% | 50% | 142 | 36% | 197.8 |
| 10-BT-8\_S12\_L001\_R1\_001 | 78.6% | 48% | 144 | 18% | 12.6 |
| 10-BT-8\_S12\_L001\_R2\_001 | 58.1% | 49% | 144 | 27% | 12.6 |
| 10-BT-9\_S13\_L001\_R1\_001 | 82.7% | 49% | 138 | 18% | 41.2 |
| 10-BT-9\_S13\_L001\_R2\_001 | 78.2% | 49% | 138 | 27% | 41.2 |
| 10-LT-10\_S16\_L001\_R1\_001 | 70.4% | 47% | 147 | 18% | 17.8 |
| 10-LT-10\_S16\_L001\_R2\_001 | 45.9% | 49% | 147 | 27% | 17.8 |
| 10-LT-8\_S14\_L001\_R1\_001 | 86.2% | 48% | 144 | 18% | 66.5 |
| 10-LT-8\_S14\_L001\_R2\_001 | 81.8% | 49% | 144 | 27% | 66.5 |
| 10-LT-9\_S15\_L001\_R1\_001 | 78.3% | 46% | 149 | 18% | 16.8 |
| 10-LT-9\_S15\_L001\_R2\_001 | 67.0% | 48% | 149 | 27% | 16.8 |
| 15-BT-6\_S17\_L001\_R1\_001 | 81.4% | 46% | 145 | 18% | 50.4 |
| 15-BT-6\_S17\_L001\_R2\_001 | 72.9% | 48% | 145 | 27% | 50.4 |
| 15-BT-7\_S18\_L001\_R1\_001 | 79.3% | 47% | 147 | 18% | 27.3 |
| 15-BT-7\_S18\_L001\_R2\_001 | 63.5% | 48% | 147 | 27% | 27.3 |
| 15-BT-8\_S19\_L001\_R1\_001 | 71.4% | 47% | 149 | 18% | 16.9 |
| 15-BT-8\_S19\_L001\_R2\_001 | 55.9% | 49% | 149 | 36% | 16.9 |
| 15C-LT-2\_S20\_L001\_R1\_001 | 85.7% | 49% | 139 | 36% | 16.6 |
| 15C-LT-2\_S20\_L001\_R2\_001 | 78.2% | 50% | 140 | 45% | 16.6 |
| 15C-LT-3\_S21\_L001\_R1\_001 | 82.4% | 50% | 131 | 36% | 15.7 |
| 15C-LT-3\_S21\_L001\_R2\_001 | 78.9% | 50% | 132 | 45% | 15.7 |
| 15C-LT-5\_S22\_L001\_R1\_001 | 89.6% | 43% | 107 | 55% | 3.0 |
| 15C-LT-5\_S22\_L001\_R2\_001 | 74.0% | 49% | 117 | 55% | 3.0 |
| 20-BT-4\_S23\_L001\_R1\_001 | 77.7% | 45% | 143 | 36% | 3.1 |
| 20-BT-4\_S23\_L001\_R2\_001 | 56.9% | 48% | 144 | 36% | 3.1 |
| 20-BT-6\_S24\_L001\_R1\_001 | 80.8% | 45% | 148 | 27% | 21.6 |
| 20-BT-6\_S24\_L001\_R2\_001 | 64.3% | 47% | 149 | 27% | 21.6 |
| 20-BT-7\_S25\_L001\_R1\_001 | 79.7% | 46% | 144 | 36% | 40.0 |
| 20-BT-7\_S25\_L001\_R2\_001 | 75.5% | 48% | 145 | 36% | 40.0 |
| 20-LT-6\_S26\_L001\_R1\_001 | 84.9% | 45% | 147 | 18% | 24.9 |
| 20-LT-6\_S26\_L001\_R2\_001 | 70.6% | 47% | 147 | 27% | 24.9 |
| 20-LT-7\_S27\_L001\_R1\_001 | 82.1% | 47% | 146 | 18% | 26.7 |
| 20-LT-7\_S27\_L001\_R2\_001 | 69.5% | 49% | 146 | 27% | 26.7 |
| 20-LT-8\_S28\_L001\_R1\_001 | 81.4% | 47% | 147 | 18% | 24.0 |
| 20-LT-8\_S28\_L001\_R2\_001 | 66.9% | 49% | 147 | 36% | 24.0 |
| 20C-5C-BT-1\_S29\_L001\_R1\_001 | 86.7% | 45% | 145 | 27% | 27.4 |
| 20C-5C-BT-1\_S29\_L001\_R2\_001 | 83.4% | 47% | 145 | 45% | 27.4 |
| 20C-5C-BT-4\_S30\_L001\_R1\_001 | 81.1% | 48% | 146 | 18% | 15.0 |
| 20C-5C-BT-4\_S30\_L001\_R2\_001 | 74.5% | 50% | 146 | 27% | 15.0 |
| 20C-5C-BT-5\_S31\_L001\_R1\_001 | 88.0% | 52% | 138 | 36% | 48.6 |
| 20C-5C-BT-5\_S31\_L001\_R2\_001 | 86.9% | 53% | 139 | 45% | 48.6 |
| 20C-5C-LT-2\_S32\_L001\_R1\_001 | 78.1% | 50% | 138 | 36% | 1.8 |
| 20C-5C-LT-2\_S32\_L001\_R2\_001 | 69.0% | 51% | 138 | 45% | 1.8 |
| 20C-5C-LT-3\_S33\_L001\_R1\_001 | 80.6% | 50% | 140 | 27% | 29.7 |
| 20C-5C-LT-3\_S33\_L001\_R2\_001 | 72.5% | 50% | 141 | 36% | 29.7 |
| 20C-5C-LT-4\_S34\_L001\_R1\_001 | 73.3% | 48% | 146 | 18% | 22.3 |
| 20C-5C-LT-4\_S34\_L001\_R2\_001 | 47.6% | 50% | 146 | 27% | 22.3 |
| 5C-5\_S8\_L001\_R1\_001 | 83.2% | 51% | 139 | 36% | 17.2 |
| 5C-5\_S8\_L001\_R2\_001 | 82.7% | 53% | 140 | 45% | 17.2 |
| 5C-7\_S9\_L001\_R1\_001 | 89.5% | 47% | 143 | 18% | 70.9 |
| 5C-7\_S9\_L001\_R2\_001 | 85.6% | 48% | 143 | 27% | 70.9 |
| 5C-8\_S10\_L001\_R1\_001 | 82.2% | 49% | 142 | 18% | 36.8 |
| 5C-8\_S10\_L001\_R2\_001 | 76.0% | 50% | 143 | 36% | 36.8 |

×

#### General Statistics: Columns

Uncheck the tick box to hide columns. Click and drag the handle on the left to change order.

Show All
Show None

| Sort | Visible | Group | Column | Description | ID | Scale |
| --- | --- | --- | --- | --- | --- | --- |
| || |  | FastQC | % Dups | % Duplicate Reads | `percent_duplicates` | None |
| || |  | FastQC | % GC | Average % GC Content | `percent_gc` | None |
| || |  | FastQC | Length | Average Sequence Length (bp) | `avg_sequence_length` | None |
| || |  | FastQC | % Failed | Percentage of modules failed in FastQC report (includes those not plotted here) | `percent_fails` | None |
| || |  | FastQC | M Seqs | Total Sequences (millions) | `total_sequences` | read\_count |

Close

## FastQC

FastQC is a quality control tool for high throughput sequence data, written by Simon Andrews at the Babraham Institute in Cambridge.

### Sequence Quality Histograms

The mean quality value across each base position in the read. See the FastQC help.

loading..

---

### Per Sequence Quality Scores

The number of reads with average quality scores. Shows if a subset of reads has poor quality. See the FastQC help.

loading..

---

### Per Base Sequence Content

The proportion of each base position for which each of the four normal DNA bases has been called. See the FastQC help.

Click a heatmap row to see a line plot for that dataset.

##### *rollover for sample name*

Position: -

%T: -

%C: -

%A: -

%G: -

---

### Per Sequence GC Content

The average GC content of reads. Normal random library typically have a roughly normal distribution of GC content. See the FastQC help.

Percentages
Counts

loading..

---

### Per Base N Content

The percentage of base calls at each position for which an N was called. See the FastQC help.

loading..

---

### Sequence Length Distribution

The distribution of fragment sizes (read lengths) found. See the FastQC help.

loading..

---

### Sequence Duplication Levels

The relative level of duplication found for every sequence. See the FastQC help.

loading..

---

### Overrepresented sequences

The total amount of overrepresented sequences found in each library. See the FastQC help for further information.

loading..

---

### Adapter Content

The cumulative percentage count of the proportion of your library which has seen each of the adapter sequences at each position. See the FastQC help. Only samples with ≥ 0.1% adapter contamination are shown.

loading..

**MultiQC v0.9 (b'0d7f54d')**
- Written by Phil Ewels,
available on GitHub.

This report uses HighCharts,
jQuery,
jQuery UI,
Bootstrap,
chroma.js,
FileSaver.js and
clipboard.js.

×

### Plot Table Data

Select Column

Select Column

Please select two table columns.

Close

×

### Regex Help

Toolbox search strings can behave as regular expressions (regexes). Click a button below to see an example of it in action. Try modifying them yourself in the text box.

`^` (start of string)
`$` (end of string)
`[]` (character choice)
`\d` (shorthand for `[0-9]`)
`\w` (shorthand for `[0-9a-zA-Z_]`)
`.` (any character)
`\.` (literal full stop)
`()` `|` (group / separator)
`*` (prev char 0 or more)
`+` (prev char 1 or more)
`?` (prev char 0 or 1)
`{}` (char num times)
`{,}` (count range)

```
samp_1
samp_1_edited
samp_2
samp_2_edited
samp_3
samp_3_edited
prepended_samp_1
tmp_samp_1_edited
tmpp_samp_1_edited
tmppp_samp_1_edited
#samp_1_edited.tmp
samp_11
samp_11111
```

See regex101.com for a more heavy duty testing suite.

Close
